# Social Support and Loneliness among Chinese Rural-to-Urban Migrant Children: A Moderated Mediation Analysis of the Roles of Social Competence and Stress Mindset

**DOI:** 10.3390/ijerph192315933

**Published:** 2022-11-29

**Authors:** Luxi Chen, Fang Yang

**Affiliations:** 1 Centre for Family and Population Research, National University of Singapore, Singapore 117570, Singapore; 2 Department of Social Work, School of Sociology and Political Science, Shanghai University, Shanghai 200444, China

**Keywords:** rural-to-urban migrant children, social support, loneliness, social competence, stress mindset, challenge, threat, China

## Abstract

Social support has been an important social-contextual protective factor against loneliness. However, how individual-level protective factors, such as social competence and a positive stress mindset, may jointly influence the relationship between social support and loneliness is less known. This study examined to what extent the link between social support and loneliness would be mediated by social competence and moderated by stress mindset among migrant children. In total, 198 rural-to-urban migrant children aged 10–14 years (56.1% girls) in Beijing, China, completed a set of self-reported questionnaires. A moderated mediation analysis was performed. We found that perceived social support was associated with a lower level of loneliness, and this association was significant only for migrant children holding a positive stress mindset (indicated by a high ratio of the stress-is-a-challenge mindset to the stress-is-a-threat mindset). Notably, across children with different stress mindsets, perceived social support was indirectly related to a lower level of loneliness through greater social competence. Our findings suggest that social competence and a stress-is-a-challenge mindset are important individual-level protective factors for migrant children to overcome loneliness. Social competence can carry the effect of social support, and a stress-is-a-challenge mindset can optimize the effect of environmental resources on mental health.

## 1. Introduction

With rapid industrialization and economic growth, an increasing number of rural residents have migrated to urban areas within the country to pursue higher education or seek better employment opportunities over the past decades [1]. There are approximately 756 million internal migrants (i.e., those who move within national borders) around the globe [2]. Nowadays, the largest rural-to-urban migration occurs in developing countries, particularly China [3] and India [4]. Growing internal migration has also been observed in other Asian countries [5] such as Indonesia [6] and Siri Lanka [7]. For example, the latest National Bureau of Statistics (NBS) annual survey documented that the number of rural-to-urban workers in China increased to 292.5 million in 2021 [3]. The expanded internal migration has brought a large number of children from rural to urban areas. According to the United Nations Children’s Fund (UNICEF) 2018 report, there were 34.3 million rural-to-urban migrant children and adolescents under 18 in China, accounting for 12.8% of the children and adolescence in the nation [8].

During the transition from rural to urban areas, migrant children face a variety of social, developmental, economic, health, and academic challenges in their new environments [9]. The international literature suggested that migration has negative impacts on children’s socioemotional development and mental health [10]. For instance, rural-to-urban migrant children in China experience numerous mental health issues such as anxiety, depression, and loneliness (for reviews, see [9,11]). 

Loneliness, an unpleasant emotional response to perceived social adversities, is a mental health issue that migrant children often experience due to acculturative stressors (i.e., the stressors that emerge during the process of adapting to new cultures), discrimination, social exclusion, or social isolation [12,13,14]. For instance, rural-to-urban migrant children in China reported greater loneliness than their local counterparts in urban areas [13,15]. As such, it is critical to explore the environmental and individual-level protective factors to facilitate this young vulnerable population to adapt to their new environments and achieve more positive psychological outcomes. 

Social support has been recognized as an important social-contextual protective factor against loneliness [16]. The positive effect of social support on health and well-being emerges as early as childhood and adolescence [17] and continues across the human life span [18]. Nonetheless, the mechanism regarding how individual-level protective factors may influence the effect of environmental resources on loneliness has been less investigated. Social competence generally refers to the skills needed for successful adaptation to social situations [19]. The concept of stress mindset was proposed to define the meta-cognitive belief about the nature of stress as either enhancing or debilitating [20,21]. Social competence and stress mindset are important psychological resources that are related to one’s responses to social adversities but have not been jointly investigated in the link between social support and loneliness. 

Therefore, we sought to fill the gap in understanding to what extent social competence and stress mindset may account for or influence the well-established relationship between social support and loneliness among migrant children.

### 1.1. Social Contexts of Rural-to-Urban Migrant Children in China

Chinese rural-to-urban migrant children are defined as children and adolescents aged 6 to 18 years old who migrate with one or both of their working parents from rural to urban areas in China. Early adolescents aged between 10 and 14 years accounted for the largest group (33.7%) of rural-to-urban migrant children in Beijing, China [22]. Adolescence is a sensitive stage of development characterized by physical, cognitive, social, and emotional maturing which provides a foundation for future health [23]. Early adolescents (i.e., 10- to 14-year-olds) usually encounter greater challenges and show more problems during social interactions and health development [23,24]. Indeed, migrant young adolescents face various stressful life events and are vulnerable to mental health issues such as depression, anxiety, and loneliness [25,26,27].

The household registration (i.e., hukou) system in mainland China has linked people’s access to public services (like hospitals and schools) to their registered residency status. The hukou of Chinese rural-to-urban migrant children is usually in rural areas or places of origin outside their urban place of residence. Most of the migrant children in Beijing are enrolled in private migrant schools that are typically located on the outskirts of the city with limited access to educational and health resources [28]. Migrant children isolated in private migrant schools are exposed to more economic difficulties, academic challenges, and social adversities including interpersonal relationships and peer victimization [29]. Consequently, these children suffer from more emotional symptoms such as depression and loneliness compared with ordinary children in urban areas [15,30] and other migrant children who were enrolled in public schools [31]. 

Hence, our work focused on rural-to-urban migrant young adolescents aged between 10 and 14 years, without an urban hukou, and enrolled in private migrant schools. Investigating the interplay between environmental and individual-level protective factors against mental health issues in this very vulnerable subgroup of migrant children is an essential step to understanding how to promote the mental health of migrant children.

### 1.2. Social Support and Loneliness

Loneliness is an unpleasant feeling caused by the discrepancy between actual and desired social relationships, either in quantity or quality [32]. Loneliness has been a prevalent mental health issue among Chinese rural-to-urban migrant children due to perceived discrimination [13], social exclusion [12], and social isolation [33]. 

Support from the family, peers, teachers, and the community provides migrant children with resources to deal with social adversities. Seeking and utilizing social support can help vulnerable children broaden their social networks, increase their social connections, get closer to their desired social relationships, and eventually experience less loneliness [34,35]. The important role of social support in mental health (e.g., increased life satisfaction and reduced loneliness) has been found in various groups of vulnerable children resulting from internal migration in China, including rural-to-urban migrant children [33,35,36,37] and left-behind children (who remain in rural areas while their parents have moved to urban areas) [34,38,39,40]. 

However, how the linkage between social support and loneliness among migrant children may be influenced by their personal strengths and competence is still unclear. Therefore, the present study aimed to explore the roles of relevant psychological resources, particularly social competence and stress mindset, in the relationship between social-contextual resources (e.g., social support) and mental health (e.g., loneliness).

### 1.3. Potential Roles of Social Competence and Stress Mindset in the Relationship between Social Support and Loneliness 

The present research was guided by the resilience framework, which emphasizes the importance of utilizing personal strengths and resources to counteract contextual risks and maintain or improve one’s health [41,42]. Resilience is broadly defined as the process of using an individual’s strengths, competencies, and resources to overcome adversities [42,43,44]. Some studies on left-behind children in China showed that resilience mediated the positive effect of social support on mental health [38], but others found that resilience moderated the association between social support and mental health, with the association being stronger for left-behind children with lower resilience [34]. Among Chinese rural-to-urban children, resilience has been found to mitigate the impact of environmental risks (e.g., social adversities) on depressive symptoms [25].

Although the literature on resilience has highlighted the importance of employing personal competence and resources to deal with environmental risks, very few studies have investigated the exact roles of different strengths and competencies in the relationship between environmental resources and mental health. In the acculturation context, social competence and stress mindset are related to coping effectiveness and psychological well-being but have been rarely examined together in the association between social support and loneliness. 

#### 1.3.1. The Potential Mediating Role of Social Competence in the Association between Social Support and Loneliness 

According to Coll et al.’s [45] integrative model for developmental competencies in minority children, the development of social competence is influenced by acculturation, socialization, and social environment (e.g., family and schools). Social support is deemed an important resource for vulnerable children to obtain knowledge and skills to deal with social adversities. In other words, seeking social support can enhance social competence. Indeed, social support has predicted greater social competence in a wide range of young populations around the world such as Latino children [46], African American preschoolers [47], and racial minorities and majorities in Italy [48]. Social support can also facilitate Chinese rural-to-urban migrant children to adapt to urban cultures and improve their social competence [49].

Moreover, social competence during childhood can predict fewer internalizing symptoms such as feelings of loneliness during late adolescence among typically developing children in urban areas in China [50]. Social competence was further found to mediate the association between peer relationships and loneliness in Chinese children [51]. It is reasonable to expect that the relation of social support to a lower level of loneliness among rural-to-urban migrant children may also be mediated by their enhanced social competence. Nevertheless, this hypothesis requires empirical examination. 

#### 1.3.2. The Potential Moderating Role of Stress Mindset in the Association between Social Support and Loneliness 

Recently, growing attention has been paid to the essential role of a positive stress mindset in promoting functional stress coping and psychological well-being. Crum and colleagues [20,21] proposed the concept of stress mindset to conceptualize the meta-cognitive belief that stress has an enhancing nature (i.e., a stress-is-enhancing mindset) or a debilitating nature (i.e., a stress-is-debilitating mindset). Empirical studies have demonstrated that a stress-is-debilitating mindset can aggravate mental health issues, while a stress-is-enhancing mindset can attenuate the impact of environmental risks (e.g., adversities) on mental health such as anxiety and depressive symptoms in adolescents [52] and young adults [53]. Among Chinese rural-to-urban migrant children, the buffering role of a stress-is-enhancing mindset has also been established in the relationship between environmental risks (e.g., stressful events) and psychological well-being [26,54]. 

In the present study, we follow Chen and colleagues’ [27,55,56] definition to classify stress mindset as a “stress-is-a-challenge mindset” (referred to as the belief that stress is an opportunity for personal growth and gains) and a “stress-is-a-threat mindset” (referred to as the belief that stress is a risk of damage, harm, or loss). Holding a stress-is-a-threat mindset is usually related to greater psychological distress such as more anxiety and depressive symptoms, whereas having a stress-is-a-challenge mindset can be related to fewer emotional symptoms through adaptive coping patterns in rural-to-urban migrant children [27] and university students [55,56]. 

This line of research has suggested that having the right stress mindset can counteract the negative impacts of environmental risks (e.g., adverse life events) on mental health across vulnerable and typically developing populations. Nonetheless, whether stress mindset can also moderate the positive effect of environmental resources (e.g., social support) on mental health is still unknown.

People with a stress-is-a-challenge mindset tend to engage in a challenge state across situations, which leads to biological, cognitive, emotional, and behavioral responses in an approach-oriented manner [57], such as greater cognitive flexibility [21], better working memory [55,58], and more functional coping patterns [27,56]. By contrast, individuals with a stress-is-a-threat mindset tend to engage in a threat state, which leads to responses in an avoidance-oriented manner [57], such as debilitated cognitive processes [20,21,55,56] and dysfunctional coping patterns [27,58]. Thus, we expected that migrant children who have a stress-is-a-challenge mindset would respond more proactively to the available support in their environments and acquire knowledge and learn social skills more efficiently through seeking and utilizing social support. Contrariwise, it was expected that migrant children who hold a stress-is-a-threat mindset may avoid the new environment (including social-contextual resources and risks) and may not make good use of the available support to enhance their social competence. In sum, we hypothesized that migrant children’s stress mindset would moderate the effect of social support on social competence, with the effect being stronger for children with a stress-is-a-challenge mindset but weaker for children with a stress-is-a-threat mindset. 

Furthermore, migrant children with a positive stress mindset are less likely to suffer from emotional symptoms such as anxiety, tension, and depression, despite their adverse experiences [26,27,54]. Experimental data showed that when exposed to positive stimuli in challenging situations, having the right mindset to focus on the enhancing nature of stress can direct people’s attentional processes toward the positive stimuli and produce sharper increases in positive affect, whereas a negative mindset that focuses on the debilitating nature of stress led to negative biases and worse cognitive and affective outcomes [21]. Thus, it is plausible that migrant children with a stress-is-a-challenge mindset may rapidly direct their attentional processes toward the positive stimuli in their environments (e.g., available support) and more efficiently ease their feelings of loneliness. Migrant children with a stress-is-a-threat mindset may be preoccupied with negative thoughts and negative emotions and tend to bias their attention toward negative stimuli rather than positive stimuli in their environments. As a result, these children may not immediately alleviate their feelings of loneliness in response to the available support. In short, we hypothesized that migrant children’s stress mindset may moderate the direct association between social support and loneliness, with the effect being stronger for children with a stress-is-a-challenge mindset but weaker for children with a stress-is-a-threat mindset.

Taken together, it is necessary to examine to what extent the direct and indirect associations between social support and loneliness may differ across migrant children holding different stress mindsets.

### 1.4. The Present Study 

**Figure 1:**
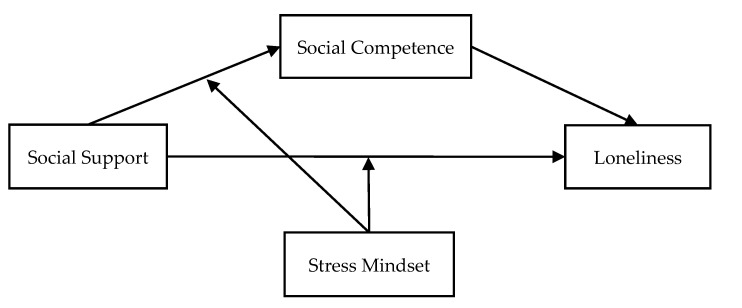
The proposed moderated mediation model.

The current research aimed to fill the gap in understanding the exact roles of social competence and stress mindset in the relationship between social support and loneliness among migrant children. Based on the literature, we proposed a moderated mediation model (see Figure 1) to examine (a) the mediating role of social competence in the relationship between social support and loneliness, and (b) the moderating role of stress mindset in the direct and indirect pathways linking social support to loneliness among migrant children. The challenge–threat ratio (dividing the stress-is-a-challenge mindset by the stress-is-a-threat mindset) was employed as the indicator of a positive stress mindset, with a higher challenge–threat ratio denoting a more positive stress mindset. 

Our hypotheses included:

**Hypothesis** **1.** *Social support is related to a lower level of loneliness among migrant children.*

**Hypothesis** **2.** *Social competence mediates the relationship between social support and loneliness, such that social support is positively related to social competence, which is further negatively related to loneliness.*

**Hypothesis** **3.** *Stress mindset moderates the association between social support and social competence, with the association being stronger for migrant children who reported a higher challenge–threat ratio than those who reported a lower challenge–threat ratio.*

**Hypothesis** **4.** *Stress mindset moderates the association between social support and loneliness, with the association being stronger for migrant children who reported a higher challenge–threat ratio than those who reported a lower challenge–threat ratio.*

## 2. Materials and Methods

### 2.1. Participants and Procedure

Inclusion criteria for this study included (1) children who migrated with one or both parents from rural areas to Beijing, (2) children without a hukou in Beijing, (3) children enrolled in private migrant schools that are typically for rural-to-urban migrant children, and (4) children in early adolescence (aged 10–14 years). Through convenience sampling, 200 migrant children were recruited from Grade 4 to Grade 6 in two private migrant schools in Tongzhou and Daxing Districts in Beijing, China. One 9-year-old and one 15-year-old were excluded from our analysis because they did not meet the fourth criterion. The final sample included 198 children (56.1% were girls) aged between 10 and 14 years (*M* = 11.8; *SD* = 1.05). Among these children, 29.8% were in fourth grade, 51.0% were in fifth grade, and 19.2% were in sixth grade. 

Data were collected in 2012. Permission was obtained from the school principals and parents to conduct the study in the schools, and no parents opted out or indicated disagreement with their children’s participation in this study. All participants were informed of the purpose of this study and indicated consent. Afterward, they completed a set of validated scales. All questionnaires were administered in Chinese. The procedure and materials were approved by the school principals and the research ethics committee of the relevant research institute.

### 2.2. Measures

#### 2.2.1. Social Support

Social support was measured by the 18-item Index of the Sojourner Social Support (ISSS) Scale [59], which was developed to measure social support in an acculturation context. Migrant children reported on a 5-point Likert-type scale with “1” indicating “no one would do this”, “2” indicating “someone would do this”, “3” indicating “a few would do this”, “4” indicating “several would do this”, and “5” indicating “many would do this”. This scale included items on generic social support (e.g., “listen and talk with you whenever you feel lonely or depressed” and “accompany you to do things whenever you need someone for company”) and items on social support in the specific environments to a sojourning population (e.g., “provide necessary information to help orient you to your new surrounding” and “give you some tangible assistance in dealing with any communication or language problems that you might face”). The ISSS scale exhibited good internal reliability (Cronbach’s *α* = 0.86) in the current sample. We computed the average score of all items on this scale to indicate social support, with the higher score indicating more social support migrant children perceived.

#### 2.2.2. Social Competence

Social competence was assessed by the 6-item social subscale of the Perceived Competence Scale for Children [60]. This scale has been widely used to measure children’s sense of competence across different domains. Migrant children decided which kind of kid they were the most like (e.g., “Easy to make friends vs. Hard to make friends” and “Most kids like me vs. Most kids dislike me”), and then rated whether the description was “sort of true” or “really true”. Each item is scored from 1 to 5, where “1” indicates low perceived social competence and “5” reflects high perceived social competence. The internal reliability of this scale was acceptable (Cronbach’s *α* = 0.71) in the current sample. Scores of all items were averaged to indicate social competence, with the higher score denoting a higher level of social competence.

#### 2.2.3. Stress Mindset

Stress-is-a-threat and stress-is-a-challenge mindsets were measured by the 12-item Chinese Making Sense of Adversity Scale [61]. Migrant children reported how they interpreted their daily adversities on a 4-point Likert-type scale ranging from 1 (totally disagree) to 4 (totally agree). Eight items measured a stress-is-a-challenge mindset (e.g., “Adversity provides a good opportunity for learning”) and four items measured a stress-is-a-threat mindset (e.g., “Stress means the end of the world and I am not able to resolve it”). These two subscales have shown good validity with coping patterns and psychological health in prior studies on migrant children [27] and typically developing youth [55,56]. Scores of all relevant items for each subscale were averaged to indicate a stress-is-a-challenge mindset (Cronbach’s *α* = 0.71) and a stress-is-a-threat mindset (Cronbach’s *α* = 0.70), respectively. Given that these two types of stress mindsets may play distinct roles during the process of stress coping [27,55,56], we computed the challenge–threat ratio (dividing the stress-is-a-challenge mindset by the stress-is-a-threat mindset) to indicate a positive stress mindset. A higher challenge–threat ratio suggests a more positive stress mindset.

#### 2.2.4. Loneliness

Feelings of loneliness were measured by the Children’s Loneliness and Social Dissatisfaction Scale [62], which has been one of the most widely used measures of children’s loneliness. The 24-item scale consists of 16 items on loneliness (including 6 regular items such as “I have nobody to talk to” and 10 reversed-scoring items such as “I have lots of friends”) and 8 filler items that ask about hobbies (e.g., “I watch a lot of TV” and “I like to paint and draw”). Children rated how true each item was for them on a 5-point scale that ranges from 1 (not true at all) to 5 (always true). After reversing scoring for the relevant items, scores of the 16 loneliness items were averaged to indicate migrant children’s feelings of loneliness, with the higher score signifying a higher level of loneliness. This scale possessed adequate internal reliability (Cronbach’s *α* = 0.76) in the current sample. 

### 2.3. Statistical Analyses

The means, standard deviations, and bivariate correlations among all main variables were calculated. The PROCESS procedure [63] was performed on the IBM SPSS Statistics 26.0 to test the proposed moderated mediation model. Age and sex were controlled for in the analysis.

## 3. Results

### 3.1. Descriptive Analyses

Descriptive statistics and bivariate correlations among all main variables are displayed in Table 1. Migrant children’s feelings of loneliness were negatively associated with their perceived social support, social competence, and a stress-is-a-challenge mindset but positively associated with a stress-is-a-threat mindset. Social competence was positively correlated with perceived social support and a stress-is-a-challenge mindset but negatively correlated with a stress-is-a-threat mindset. 

### 3.2. A Moderated Mediation Analysis of the Roles of Social Competence and Stress Mindset in the Relationships between Social Support and Loneliness 

As shown in Table 2, perceived social support was associated with a lower level of loneliness. The significant interaction between social support and stress mindset on loneliness indicated that the direct association between social support and loneliness was moderated by migrant children’s stress mindset. The conditional effects are presented in Table 3. The relation of perceived social support to a lower level of loneliness was significant only for migrant children who had a positive stress mindset (i.e., a high challenge–threat ratio) but not significant for children who reported a medium or low challenge–threat ratio. 

Furthermore, the association between perceived social support and loneliness was in part mediated by migrant children’s social competence. The nonsignificant interaction between social support and stress mindset on social competence indicated that the association between social support and social competence was not moderated by migrant children’s stress mindset. Across migrant children holding different stress mindsets (i.e., different levels of challenge–threat ratio), perceived social support was related to greater social competence, which was further related to a lower level of loneliness. 

**Figure 2:**
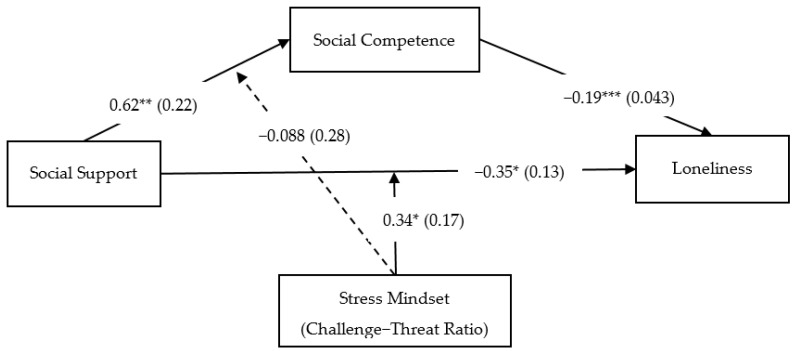
The final model: The association between social support and loneliness was mediated by migrant children’s social competence and moderated by their stress mindset (indicated by the challenge–threat ratio). Note. The challenge–threat ratio was calculated by dividing the stress-is-a-challenge mindset by the stress-is-a-threat mindset. *** *p* < 0.001. ** *p* < 0.01. * *p* < 0.05.

To sum up, the association between social support and loneliness was mediated by migrant children’s social competence and moderated by their stress mindset. All variables in this model explained 31.6% of the variations in feelings of loneliness: *F*(6, 191) = 20.3, *p* < 0.001. The final model is illustrated in Figure 2.

### 3.3. Sensitivity Analysis

To confirm the direction of the correlation between social support and social competence in our target model, a sensitivity analysis was conducted to rule out the possibility that social competence may act as the predictor rather than the outcome of perceived social support. In the alternate model, social competence was entered as the predictor and social support was entered as the mediator. The path from social competence to social support was nonsignificant (*b* = 0.15, *SE* = 0.13, *t* = 1.18, *p* = 0.24, and *95% CI* [−0.10, 0.41]), and neither the path from social competence to loneliness nor the path from social support to loneliness was statistically significant in the alternate model (*ps* > 0.050). Results suggest that our proposed mediating pathway instead of the alternated model can be supported by the data: perceived social support predicted greater concurrent social competence, which was further related to a lower level of loneliness. 

## 4. Discussion

The present work has filled the research gap by addressing the roles of social competence and stress mindset in the relationship between social support and loneliness among migrant children. Consistent with Hypotheses 1 and 4, migrant children’s stress mindset moderated the well-established relationship between social support and loneliness. The direct relation of perceived social support to a lower level of loneliness was significant only for migrant children who had a positive stress mindset (indicated by a high ratio of the stress-is-a-challenge mindset to the stress-is-a-threat mindset, namely a high challenge–threat ratio). In line with Hypothesis 2, social competence mediated the link between social support and loneliness; but contrary to Hypothesis 3, this mediating pathway did not vary by migrant children’s stress mindset. The indirect association between perceived social support and loneliness through social competence was significant across children with different stress mindsets (indicated by different levels of challenge–threat ratio). 

### 4.1. Stress Mindset Moderates the Association between Social Support and Loneliness

Despite the emerging literature on the buffering role of a positive stress mindset in the relationship between environmental risks and mental health issues [26,53,54], this was the first study that examined the moderating role of stress mindset in the link between environmental resources and mental health among migrant children. Previous research demonstrated that having the right stress mindset can attenuate the negative impacts of environmental risks (e.g., stressful events) and reduce migrant children’s mental health issues (e.g., depressive symptoms) [26,27,54]. Our findings have added to the literature by illustrating that a stress-is-a-challenge mindset can optimize the positive effect of environmental resources (e.g., social support) on migrant children’s mental health (e.g., a lower level of loneliness). 

Crum and colleagues’ prior experiment demonstrated that, when exposed to positive stimuli, participants who believed that stress has an enhancing nature experienced a sharper increase in positive affect than their counterparts who believed that stress has a debilitating nature [21]. We took a further step to discover that, when provided with social support, migrant children with a stronger stress-is-a-challenge mindset benefited directly from perceived social support to a greater extent and were less likely to experience loneliness than their counterparts with a stress-is-a-threat mindset.

Migrant children with a stress-is-a-challenge mindset tend to focus on the opportunities for learning, personal growth, and gains inherent in stress. They may be able to rapidly direct their attention toward positive information in their environments (e.g., social support) and efficiently evoke positive emotional responses to the available support. Moreover, having a positive attitude toward stress enables individuals to respond to the environment in an approach manner cognitively, emotionally, and behaviorally, such as showing greater cognitive flexibility [21], adopting more approach coping strategies like support-seeking and problem-solving [27], displaying greater coping flexibility [55,56], and experiencing more positive affect like excitement and happiness [21,55]. These processes may further facilitate migrant children to make better use of the available support in their social networks and consequently feel less lonely. Contrariwise, migrant children who hold a stress-is-a-threat mindset tend to interpret adversities as risks of loss and harm. A stress-is-a-threat mindset biases their attentional processes toward negative stimuli rather than positive stimuli in their environments, so these children may not proactively respond to the available support. Instead, they tend to react to the environment in an avoidance manner, such as engaging in an avoidance coping pattern like behavioral disengagement, denial, and self-blame [27], showing worse cognitive flexibility [21] and impeded working memory [55,58], and experiencing more negative affect like tension and anxiety [55,58]. When being overwhelmed by negative thoughts, negative affect, avoidance behaviors, as well as attentional and interpretational biases, migrant children with a stress-is-a-threat mindset may not swiftly reduce their feelings of loneliness. 

Taken together, fostering a stress-is-a-challenge mindset and reducing a stress-is-a-threat mindset may empower migrant children to benefit more from environmental resources such as social support and improve their psychological health.

### 4.2. Social Competence Mediates the Association between Social Support and Loneliness

We further investigated to what extent migrant children’s social competence may explain the association between perceived social support and loneliness, and whether this indirect association would be moderated by migrant children’s stress mindset. We discovered that migrant children’s social competence in part mediated the relationship between social support and loneliness, and remarkably, this indirect effect did not vary by migrant children’s stress mindset. 

Social competence has been found in previous studies to mediate the relationship between interpersonal relationships and loneliness among typically developing children in China [51]. We established a similar pattern in rural-to-urban children in China: perceiving more social support was related to their greater concurrent social competence. Indeed, the development of the social competence of minority children is largely shaped by their social environments, socialization, and acculturation [45]. Social support is an important environmental protective factor that can equip children with knowledge, skills, and resources to deal with adversities [49]. During the processes of seeking, receiving, and utilizing social support, migrant children learn to socialize with people around them and adapt to their new social environments, and consequently, they may experience less social exclusion and isolation, and eventually feel less lonely. 

More importantly, although the direct effect of social support on loneliness was significant only for migrant children with a positive stress mindset (indicated by a high challenge–threat ratio), social support can still indirectly protect all migrant children from loneliness by improving their social competence regardless of their stress mindset. Across migrant children holding different stress mindsets (indicated by different levels of challenge–threat ratio), greater social support is beneficial for them to practice social skills and utilize knowledge and resources to deal with social adversities and ultimately reduce feelings of loneliness and achieve better mental health. 

Our findings highlighted that social competence is an important psychological resource that carries the effects of environmental resources (e.g., social support) on migrant children’s mental health.

### 4.3. Implications, Limitations, and Future Directions

Theoretically, this study has advanced the literature by illustrating the mechanism regarding how social support is linked to migrant children’s mental health through social competence, and moreover, how these pathways may be moderated by stress mindset. As discussed in the previous section, this was the first investigation that examined the moderating role of stress mindset in the linkages among social support, social competence, and loneliness. Our findings shed light on resilience research by revealing that social competence and a stress-is-a-challenge mindset are important child-level protective factors against loneliness for migrant children. In particular, social competence can carry the effect of social support, and a stress-is-a-challenge mindset can moderate the effect of social support on migrant children’s feelings of loneliness. Also, these findings have extended the research on stress coping by suggesting that a positive stress mindset not only buffers the negative impact of environmental risks but also optimizes the positive effect of environmental resources on mental health. 

The present research has also provided some practical implications for policymakers, educators, and social workers. Firstly, creating a favorable environment and expanding access to environmental resources, services, and support for migrant children is an essential step to promoting the mental health of this vulnerable young population. Furthermore, improving migrant children’s psychological resources can facilitate them to respond proactively to the available environmental resources and achieve better mental health. Previous research suggests that resilience-based intervention programs can effectively train personal strengths, competence, resources, and positive attitudes toward adversities among migrant children, and ultimately, enhance their psychological well-being [64]. Social competence and a stress-is-a-challenge mindset are crucial individual-level protective factors relevant to the acculturation context and should be incorporated into resilience-based intervention programs. By enhancing social competence and boosting a stress-is-a-challenge mindset, migrant children will be equipped with greater resources and abilities to make better use of environmental resources and mitigate the negative impacts of environmental risks, so as to achieve more positive psychological outcomes. 

We also see some limitations in the present investigation that can guide future research. First, some migration-related factors (such as the number of years they had stayed in the destination, family socioeconomic status, living arrangement, and home environment) are related to social support and adaptation to the environment but were not captured in this study. Future research should take into account migration-related variables and socioeconomic demographics in order to understand or rule out their effects on the linkages among environmental resources, personal strengths, and mental health. The second limitation had to do with the on-site self-report measures. The accuracy of the data can be threatened by self-reporting biases such as social desirability and recall bias. Future studies should collect data from different informants (such as peers, teachers, and parents) on children’s social support and social competence and should capture migrant children’s emotional states more precisely using implicit measures or biomarkers. Finally, although a sensitivity analysis was performed to confirm the direction of the correlation between social support and social competence in the context of studying loneliness, cross-sectional data were unable to establish causality. Future research will benefit from using an experimental or intervention study to test the cause-and-effect relationships among social support, social competence, stress mindset, and loneliness. It is also necessary to employ a longitudinal design to examine the long-term effects of environmental and child-level protective factors on migrant children’s psychological well-being later in life. 

## 5. Conclusions

The present research has addressed the gap in understanding the roles of social competence and stress mindset in the link between social support and loneliness. On the one hand, the relation of social support to a lower level of loneliness can be moderated by migrant children’s stress mindset, with the direct association being stronger for children with a more positive stress mindset (indicated by a higher ratio of the stress-is-a-challenge mindset to the stress-is-a-threat mindset). On the other hand, social support can be indirectly related to a lower level of loneliness through greater social competence, and this indirect association did not vary by migrant children’s stress mindset. Our findings suggest that a stress-is-a-challenge mindset is a child-level protective factor that empowers migrant children to benefit from environmental resources (e.g., social support) and reduce feelings of loneliness to a greater extent. Social competence is another important psychological resource that can carry the protective role of social support against loneliness across migrant children holding different stress mindsets. The present research has advanced the literature on resilience by highlighting the importance of utilizing individual-level strengths and resources to not only counteract the negative impacts of environmental risks but also optimize the positive effects of environmental resources on mental health. We recommended the incorporation of a stress-is-a-challenge mindset and social competence into future resilience-based intervention programs to promote the mental health of migrant children.

## Figures and Tables

**Table 1:** Descriptive statistics and bivariate correlations among all main variables.

	1 Social Support	2 Social Competence	3 Challenge Mindset	4 Threat Mindset	5 Challenge–Threat Ratio	6 Loneliness	7 Sex (Girl)	8 Age
1	-							
2	0.49 ***	-						
3	0.16 *	0.23 **	-					
4	−0.018	−0.15 *	−0.19 **	-				
5	0.097	0.22 **	0.64 ***	−0.81 ***	-			
6	−0.32 ***	−0.45 ***	−0.32 ***	0.27 ***	−0.37 ***	-		
7	−0.003	−0.035	−0.18 *	0.086	−0.13	0.19 **	-	
8	0.083	−0.029	0.068	0.012	0.043	0.18 *	0.084	-
*M*	2.61	3.53	2.79	2.05	1.50	2.06	56.1%	11.7
*SD*	0.70	0.83	0.52	0.57	0.62	0.50	-	1.14

Note. Challenge mindset = a stress-is-a-challenge mindset. Threat mindset = a stress-is-a-threat mindset. *** *p* < 0.001. ** *p* < 0.01. * *p* < 0.05.

**Table 2:** Results of the moderated mediation model.

	*b*	*SE*	*t*	*95% CI*
**Mediator: Social Competence**				
Social Support	0.62 **	0.22	2.82	[0.19, 1.06]
Stress Mindset (CT Ratio)	−0.35	0.74	0.47	[−1.81, 1.11]
Social Support × CT Ratio	−0.088	0.28	−0.31	[−0.65, 0.47]
Sex (girl)	−0.045	0.11	−0.43	[−0.25, 0.16]
Age	−0.057	0.046	−1.24	[−0.15, 0.034]
**Outcome: Loneliness**				
Social Support	−0.35 *	0.13	−2.59	[−0.61, −0.082]
Stress Mindset (CT Ratio)	−0.37	0.44	−0.84	[−1.24, 0.50]
Social Support × CT Ratio	0.34 *	0.17	2.01	[0.007, 0.67]
Social Competence	−0.19 ***	0.043	−4.55	[−0.28, −0.11]
Sex (girl)	0.11	0.065	1.61	[−0.024, 0.23]
Age	0.080 **	0.028	2.87	[0.025, 0.14]

Note. CT ratio = challenge–threat ratio (calculated by dividing the stress-is-a-challenge mindset by the stress-is-a-threat mindset) as an indicator of a positive stress mindset. *** *p* < 0.001. ** *p* < 0.01. * *p* < 0.05.

**Table 3:** The conditional association between social support and loneliness.

Social Support to Loneliness	*Effect*	*SE*	*95% CI*
**Direct Association**			
High CT Ratio	−0.13	0.059	[−0.24, −0.013]
Medium CT Ratio	−0.080	0.052	[−0.18, 0.022]
Low CT Ratio	−0.057	0.062	[−0.18, 0.066]
**Indirect Association through Social Competence**			
High CT Ratio	−0.11	0.029	[−0.17, −0.058]
Medium CT Ratio	−0.11	0.032	[−0.18, −0.054]
Low CT Ratio	−0.11	0.029	[−0.17, −0.059]

Note. CT ratio = challenge–threat ratio (calculated by dividing the stress-is-a-challenge mindset by the stress-is-a-threat mindset) as an indicator of a positive stress mindset.

## Data Availability

The data presented in this study are available on request from the corresponding author.
